# Antimicrobial use in patients with confirmed COVID-19 infection in the Philippines: a cross-sectional study

**DOI:** 10.5365/wpsar.2023.14.2.999

**Published:** 2023-06-24

**Authors:** Roanne J Dominguez, Nicole A Domingo-Cereno, Rosemarie T Josue-Dominguez

**Affiliations:** a Saint Louis University Hospital of the Sacred Heart, Baguio City, Philippines.

## Abstract

**Objective:**

The ongoing coronavirus disease (COVID-19) pandemic is exacerbating optimal antibiotic stewardship and the promotion of bacterial resistance due to the over-prescribing of antibiotics for patients with COVID-19. This study aimed to determine the prevalence of antibiotic therapy in patients with COVID-19 infection and explore the association of antibiotic prescribing with patients’ demographics and clinical characteristics.

**Methods:**

A retrospective analytical cross-sectional study was conducted at a tertiary hospital and training institution in Baguio City, the Philippines from March 2020 to March 2021. Univariate and multivariable logistic regression was used to compare COVID-19 patients who were prescribed antibiotics with those who were not.

**Results:**

Of the 157 patients hospitalized with COVID-19 infection, 90 (57.3%) received antibiotics, with only three (1.9%) having confirmed bacterial coinfection. Among those prescribed antibiotics, azithromycin was the most frequently prescribed antibiotic (43.3%), followed by ceftriaxone (33.1%), piperacillin-tazobactam (15.3%), ceftazidime (5.1%), moxifloxacin (1.3%), amikacin (0.6%), ampicillin and sulbactam (0.6%), cefuroxime (0.6%), metronidazole (0.6%) and penicillin (0.6%). Antibiotic use was associated with factors such as having bilateral infiltrates on chest X-ray, the severity of COVID-19 infection and high white blood cell counts.

**Discussion:**

Antibiotic use was high among patients with confirmed COVID-19 despite a low prevalence of confirmed bacterial coinfection. This may be due to the similarities in the clinical manifestations of both viral and bacterial infections. Judicious use of antibiotics in the treatment of COVID-19, as well as other viral infections (for example, influenza), is required to prevent antibiotic resistance in accordance with the principles of antimicrobial stewardship.

Coronavirus disease (COVID-19), caused by severe acute respiratory syndrome coronavirus 2 (SARS-CoV-2), was first documented in Wuhan, Hubei Province, China in December 2019. (*1*) According to the World Health Organization (WHO), as of April 2023, there have been 762 million COVID-19 cases with over 6.8 million deaths worldwide. In the Philippines, there have been over 4 million confirmed cases with over 66 000 deaths. (*2*) The global incidence has steadily declined in 2023 after a peak in December 2022. (*2*) However, severe and critical disease remains a concern; in one study from the United States of America, 5.3% of cases infected with the Omicron variant were hospitalized, with 3% requiring oxygen. (*3*) Internationally (*4*) and in the Philippines, (*5*) severe and critical infections usually affect older patients and those with multiple comorbidities.

Respiratory viral infections are a risk factor for bacterial coinfections, which may increase disease severity and mortality. (*6*) Bacterial coinfections are defined as suspected bacterial pneumonia in addition to COVID-19 within 48–72 hours of hospital admission for COVID-19, (*7*) and are relatively common in patients with severe and critical disease. (*8*) Secondary bacterial infections are defined as suspected bacterial pneumonia after 72 hours of hospitalization for COVID-19, (*7*) and are diagnosed when patients present with the symptoms and signs of pneumonia and a pathogen is isolated from sputum, blood, endotracheal aspirate or bronchoalveolar lavage cultures following admission. (*1*) There are limited cues for differentiating bacterial and viral respiratory infections.

Despite the viral origin of COVID-19, physicians tended to start treatment with antibiotics since cough, fever and infiltrates on chest imaging are markers of bacterial community-acquired pneumonia requiring antibiotics. (*9*) The uncertainty of the COVID-19 pandemic and the absence of antiviral treatments with proven efficacy probably also contributed to the widespread and excessive use of antibiotics, (*10*) especially in the first year of the pandemic. This prescriber behaviour threatens antimicrobial stewardship, which is defined as “an organizational or healthcare-system-wide approach to promoting and monitoring judicious use of antimicrobials to preserve their future effectiveness.” (*11*) WHO recommends that antimicrobials be used for severe COVID-19 cases at increased risk for secondary bacterial infection and death. (*12*)

The main objective of this study is to describe antibiotic use in patients with confirmed COVID-19 infection at a tertiary hospital in Baguio City, Philippines. More specifically, the study aims to: (1) determine the prevalence of antibiotic use in patients with confirmed COVID-19 infection; (2) verify the prevalence of bacterial coinfection; (3) ascertain the most frequently prescribed antibiotics; and (4) explore the associations of variables with antibiotic use, specifically, age, sex, comorbidities, severity of COVID-19 infection, chest X-ray findings, white blood cell count, differential count, procalcitonin, blood culture, and sputum and endotracheal aspirate culture.

## Methods

### Study design

A retrospective analytical cross-sectional study was conducted at a tertiary hospital and training institution in Baguio City, Philippines.

### Study population

All adult patients (≥ 19 years old) with mild, moderate, severe and critical confirmed COVID-19 infection who were seen, diagnosed and eventually hospitalized from March 2020 to March 2021 were included in the study. Patients who were asymptomatic, regardless of the presence or absence of comorbidities, as well as patients who developed hospital-acquired infection during the course of their hospital stay, were excluded.

### Data collection

Charts of confirmed COVID-19 patients who met the inclusion criteria were reviewed. Data collected were: antibiotic usage (use or non-use, type of antibiotic); age (19–59 years old or ≥ 60 years old); presence or absence of comorbidities; disease severity (mild, moderate, severe or critical); results of chest X-ray (normal, unilateral infiltrates or bilateral infiltrates); white blood cell count (< 5000, 5000–10 000 or > 10 000); differential count (neutrophilia or lymphocytosis); procalcitonin (≤ 2 ng/mL or > 2 ng/mL); and blood, sputum and/or endotracheal aspirate cultures (with or without growth) (**Table 1**).

**Table 1:** Characteristics of hospitalized COVID-19 cases at a tertiary hospital in Baguio City, the Philippines, March 2020 to March 2021 (*n* = 157)

Characteristic	Number	%
**Age (years)**		
19–59	106	67.5
≥ 60	51	32.5
**Sex**		
Male	96	61.1
Female	61	38.9
**Comorbidities^a^**		
Yes	97	61.8
No	46	29.3
**Severity**		
Mild	64	40.8
Moderate	36	22.9
Severe	50	31.8
Critical	7	4.5
**Chest X-ray**		
Normal	80	51
Unilateral	20	12.7
Bilateral	57	36.3
**White blood cell count^a^**		
< 5 000	28	17.8
5000–10 000	99	63.1
> 10 000	26	16.6
**Differential count^a^**		
Neutrophilia	149	94.9
Lymphocytosis	4	2.5
**Procalcitonin**		
≤ 2 ng/mL	77	49
> 2 ng/mL	5	3.2
Not requested	75	47.8
**Bacterial coinfection**		
Yes	3	1.9
No	77	49
Not requested	77	49

^a^ Values are missing from some patients for comorbidities (*n* = 14), white blood cell count (*n* = 4) and differential count (*n* = 4).

The severity classification of patients with COVID-19 was based on the Unified COVID-19 Algorithms (**Table 2**). (*13*) The comorbidities included were diabetes, hypertension, coronary artery disease, rheumatic heart disease, asthma, chronic obstructive pulmonary disease, chronic kidney disease, cancer, arrhythmia and stroke. Patients were diagnosed with a bacterial coinfection if there was growth in culture samples conducted within the first 48 hours of admission to hospital.

**Table 2:** Severity classification of COVID-19 cases, the Philippines, 2020

Classification	Signs and symptoms
Mild	Fever, cough, diarrhoea, change in taste or smell, or fatigue; no signs of hypoxia on pulse oximetry or arterial blood gas, or pneumonia on physical examination and chest X-ray
Moderate	Symptomatic with clinical or radiographic evidence of lower respiratory tract disease (infiltrates on chest X-ray, presence of crackles) and oxygen saturation > 94% on room air
Severe	Symptomatic with oxygen saturation ≤ 94% on room air and lung infiltrates on chest X-ray
Critical	Respiratory failure not fully explained by cardiac failure or fluid overload (acute respiratory distress syndrome), septic shock or multiple organ dysfunction

*Source*: Unified COVID-19 Algorithms. (*13*)

### Data analysis

Data were encoded and analysed using SPSS v24 (IBM Corp., Armonk, NY, United States of America). Frequencies and percentages were used to describe the prevalence of antibiotic use in patients with COVID-19. To determine the association between antibiotic use and the variables of interest (age, sex, comorbidities, severity of COVID-19 infection, chest X-ray findings, white blood cell count, differential count, procalcitonin, blood culture, and sputum and endotracheal aspirate culture), univariate and multivariable logistic regression was used. Imputation of missing variables for some patients at hospital admission was considered if < 20% of values were missing, and imputation based on the expectation−maximization algorithm method was used to replace missing values. A *P*-value of < 0.05 was considered statistically significant.

## Results

**Fig. 1:**
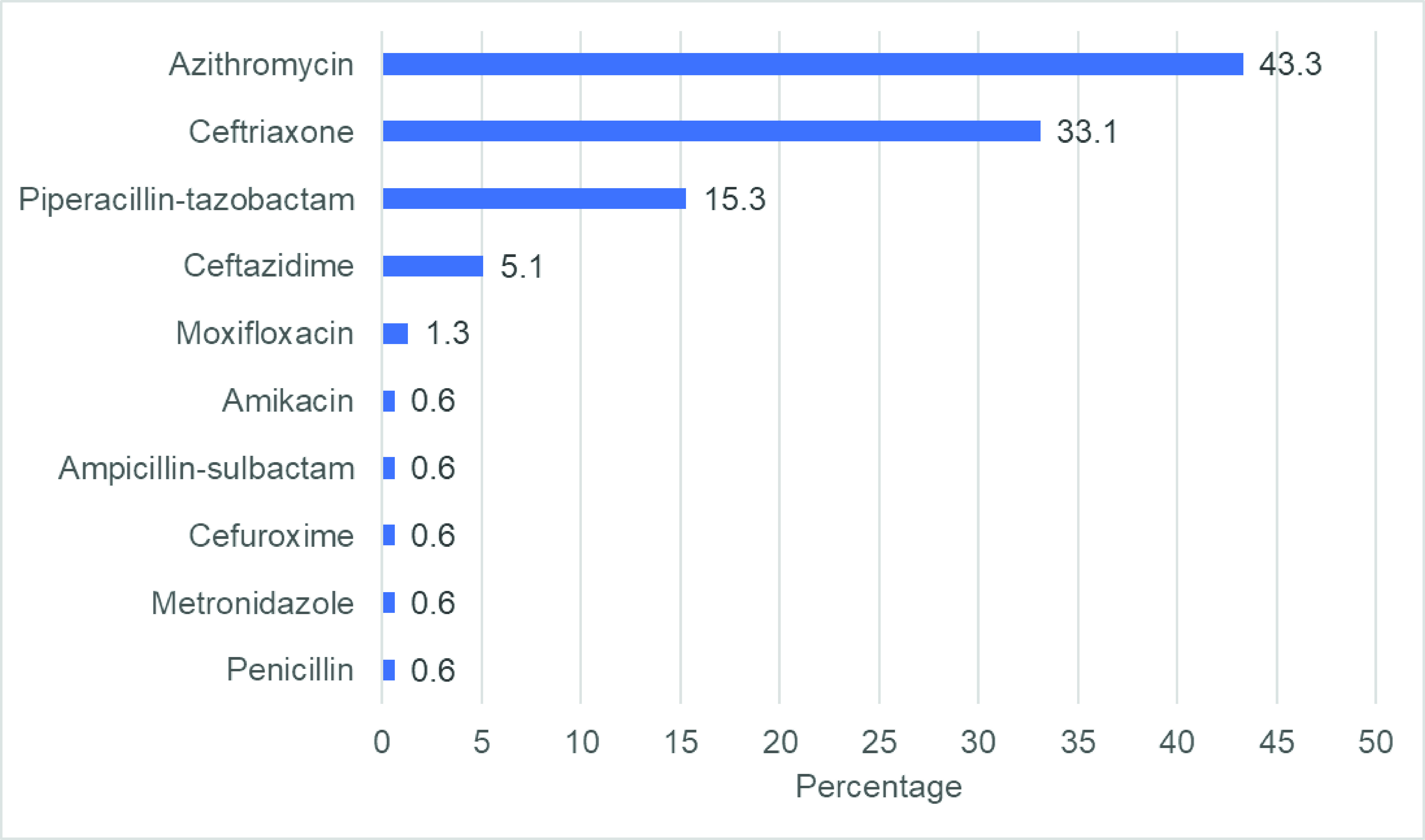
Frequency of antibiotics prescribed to hospitalized COVID-19 patients at a tertiary hospital in Baguio City, the Philippines, March 2020 to March 2021 (N = 90)

The charts were reviewed of all 157 hospitalized COVID-19 patients, of whom 90 (57.3%) received antibiotics and three (1.9%) had confirmed bacterial coinfection. Among the 90 patients who were given antibiotics, azithromycin was the most frequently prescribed antibiotic (43.3%), followed by ceftriaxone (33.1%) and piperacillin-tazobactam (15.3%) (**Fig. 1**).

[**insert** Figure 1]

There were 106 patients (67.5%) aged 19–59 years and 51 (32.5%) aged ≥ 60 years. There were more males (61.1%) than females (38.9%). Comorbidities were reported for 97 patients (61.8%). They included diabetes mellitus, hypertension, cancer, chronic kidney disease, coronary artery disease, bronchial asthma and chronic obstructive pulmonary disease. With regards to the severity of COVID-19 infection, 64 patients (40.8%) were mild, 36 (22.9%) were moderate, 50 (31.8%) were severe and seven (4.5%) were critical (**Table 1**).

Eighty patients (51.0%) had a normal chest X-ray, 20 (12.7%) had unilateral infiltrates and 57 (36.3%) had the presence of bilateral infiltrates on chest X-ray. Twenty-eight patients (17.8%) had white blood cell counts of < 5000, 99 (63.1%) had counts of 5000–10 000 and 26 (16.6%) had counts of > 10 000. Regarding differential counts, neutrophilia was noted in 149 patients (94.9%), while only four patients (2.5%) had lymphocytosis. Of the 157 patients, procalcitonin was measured in only 82 patients, of whom 77 (49%) had results of ≤ 2 ng/mL and five (3.2%) of > 2 ng/mL (**Table 1**).

Factors significantly associated with antibiotic use in multivariable analysis were: having bilateral chest X-ray infiltrates (odds ratio [OR] 48.11, 95% confidence interval [CI] 11.24–205.88, *P* < 0.001); severity of COVID-19 infection (moderate: OR: 8.98, 95% CI: 2.833–28.477, *P* < 0.001; severe: OR: 4.81, 95% CI: 1.38–16.71, *P* = 0.014; critical: OR: 0.24, 95% CI: 0.07–0.81, *P* = 0.021); and having elevated white blood cell count (5000–10 000: OR: 7.85, 95% CI: 1.28–48.29, *P* = 0.026; > 10 000: OR: 7.12, 95% CI: 1.48–34.36, *P* = 0.015) (**Table 3**).

**Table 3:** Factors associated with antibiotic use in hospitalized COVID-19 cases at a tertiary hospital in Baguio City, the Philippines, March 2020 to March 2021^a^

Factors	Univariate		Multivariable	
	OR (95% CI)	*P*	OR (95% CI)	*P*
**Age (years)**				
19–59	Ref		Ref	
≥ 60	2.0 (1.0–4.0)	**0.049**	0.3 (0.1–1.2)	**0.116**
**Sex**				
Male	Ref		Ref	
Female	0.8 (0.4–1.6)	0.644	1.7 (0.3–8.8)	0.507
**Comorbidities**				
No	Ref		Ref	
Yes	0.5 (0.2–1.0)	0.069	0.4 (0.7–3.0)	0.438
**Severity of COVID-19 infection**				
Mild	Ref		Ref	
Moderate	22.5 (9.7–52.0)	** < 0.001**	8.9 (2.8–28.4)	** < 0.001**
Severe	10.7 (4.4–25.9)	** < 0.001**	4.8 (1.3–16.7)	**0.014**
Critical	0.1 (0.0–0.2)	** < 0.001**	0.2 (0.0–0.8)	**0.021**
**Chest X-ray**				
Normal	Ref		Ref	
Unilateral	3.1 (0.5–17.2)	0.180	1.9 (0.3–11.8)	0.454
Bilateral	57.7 (16.2–206.0)	** < 0.001**	48.1 (11.2–205.8)	** < 0.001**
**White blood cell count**				
< 5 000	Ref		Ref	
5000–10 000	8.8 (2.1–36.3)	**0.003**	7.8 (1.2–48.2)	**0.026**
> 10 000	6.6 (1.8–23.6)	**0.003**	7.1 (1.4–34.3)	**0.015**
**Differential count**				
Neutrophilia	Ref		Ref	
Lymphocytosis	0.2 (0.0–2.2)	0.209	0.1 (0.0–1.8)	0.149
**Procalcitonin**				
≤ 2 ng/mL	Ref		Ref	
> 2 ng/mL	1.5 (0.1–14.2)	0.724	0.6 (0.0–11.9)	0.794
**Bacterial coinfection**				
No	Ref		Ref	
Yes	3.7 (0.3–45.9)	0.297	4.8 (0.1–127.6)	0.346

^a^ Statistically significant *P*-values (< 0.05) are in bold.

## Discussion

The prevalence of empiric antimicrobial use at this tertiary hospital in Baguio City, the Philippines was 57.3%, which is high considering that the prevalence of bacterial coinfection was 1.9%. However, similar studies have reported higher antibiotic use in patients with COVID-19 from rates of 70–90%. (*14*-*17*) In a cohort study from Wuhan, China in 2020, (*1*) all patients with laboratory-confirmed COVID-19 were given empiric antibiotic therapy. Prescribing antibiotics for COVID-19 patients was based on the WHO interim guidelines to treat for possible bacterial infection. (*18*, *19*) In two smaller studies from Jiangsu and Wuhan, antibiotics were prescribed to almost all patients. (*20*, *21*) In a study conducted by Rawson et al., (*14*) 72% of patients with COVID-19 received antimicrobial therapy, though only 8% of patients were reported to have bacterial coinfection. This may be due to difficulty ruling out bacterial coinfection during patients’ admission since viral and bacterial pneumonia have similar clinical manifestations. In a 2020 global survey of antibiotic-prescribing practices for patients with COVID-19, respondents reported that their decision to use antibiotics was based more on clinical presentation and less on laboratory or radiologic markers. (*22*) Many of these studies were from 2020, early in the COVID-19 pandemic, when antiviral treatments for COVID-19 were not available.

Almost half of the patients included in this study had mild COVID-19 infection and, therefore, as per local practice guidelines, sputum and bacterial cultures were not indicated. (*23*) This could account for the low prevalence of bacterial coinfection in this study. Rates of bacterial coinfection in patients with COVID-19 have been low, as confirmed by several studies. (*14*-*17*, *24*) In contrast, a study from Wuhan, China revealed a higher bacterial coinfection rate of 25.5% in patients admitted for COVID-19. (*25*) In a study from a secondary-care setting in the United Kingdom of Great Britain and Northern Ireland, blood cultures were positive in 3.2% of patients during the first 5 days of admission; after 5 days of confinement, the positivity rate increased to 6.1%. The same study revealed that pathogenic bacteria were identified at a higher rate (34.8%) from respiratory samples. (*26*)

Azithromycin, ceftriaxone, piperacillin-tazobactam and ceftazidime were the most commonly used antibiotics in this study. The distribution of antibiotics used follows the Philippine Clinical Practice Guidelines on the management of community-acquired pneumonia (*27*) and the antibiogram of the hospital. This finding was similar to that of a retrospective cohort study done at a COVID-19 referral hospital in the Philippines by Abad et al. (*28*) In contrast, a study from a German university hospital revealed that the most commonly used antibiotics were fluoroquinolones, carbapenems and third-generation cephalosporins; (*6*) however, this may be due to different antibiotic protocols in Europe.

The presence of bilateral pulmonary infiltrates on chest X-ray was the most significant predictor of antibiotic use in this study. Such radiologic findings increase the probability of bacterial infection. Cheng et al. (*24*) reported a similar finding in a hospital in Hong Kong Special Administrative Region (China). This study also showed that the severity of illness was associated with antibiotic use, suggesting that disease severity had a potential role in the decision to prescribe antibiotics to COVID-19 patients. Patients who are severely to critically ill develop a systemic inflammatory response that may lead to lung injury and organ dysfunction, ultimately increasing the risk of bacterial coinfection. A study by Nasir et al. (*29*) showed that patients with severe to critical COVID-19 infection on admission had 4.42 times higher risk of bacterial infection. Langford et al. (*30*) reported that the percentage of antibiotic use was especially high in patients in the intensive care unit and for those requiring mechanical ventilation. However, in a scoping review of the first 6 months of the pandemic, antibiotics were prescribed to COVID-19 patients regardless of severity of illness, with similar proportions prescribed to patients with severe or critical illness (75.4%) and patients with mild or moderate illness (75.1%). (*31*) Chedid et al. (*32*) suggested that although antibiotic treatment was more prevalent in more severe patients, half of the patients who received antibiotics were not severe, suggesting a tendency to extend indications of antibiotic therapy to non-severe patients.

Antibiotic use was also influenced by elevated white blood cell counts in the present study. COVID-19 patients usually have normal white blood cell counts. A study by Huang et al. (*18*) reported that white blood cell counts in patients with COVID-19 on admission indicated leucopenia (25%) with lymphocytic predominance (64%). Leucocytosis with neutrophilic predominance alerts physicians to the presence of bacterial coinfection. A study by He et al. (*33*) showed that antibiotic prescription was significantly more common in patients with leucocytosis. In contrast, the study by Cheng et al. (*24*) demonstrated that antibiotics were commonly ordered even if routine blood tests showed normal white blood cell count.

The limited number of patients in this study restricts the generalization of the results to a broader population, as does the lack of a comparison group, such as antibiotic prescription rates before the COVID-19 pandemic, to determine if antibiotic-prescribing habits changed or increased during the COVID-19 pandemic. Therefore, we recommend that a similar study be conducted with a larger and more diverse sample size that could include other provinces in the country to obtain a better understanding of trends in antimicrobial use in patients with confirmed COVID-19 infection.

Antibiotic use was high among patients with confirmed COVID-19 in the tertiary hospital in the Philippines during the first year of the pandemic despite a low prevalence of confirmed bacterial coinfection. Similarly, the high rates of prescribing antibiotics for COVID-19 patients were observed globally, especially in the first year of the pandemic, for both severe and non-severe cases. Factors associated with antibiotic use were radiologic evidence of bilateral infiltrates, severity of COVID-19 pneumonia and leucocytosis. The similarities in the clinical manifestations of both viral and bacterial infections may have contributed to the increased use of antimicrobials during this period, as well as there being no antiviral treatment for COVID-19 available at that time. Judicious use of antibiotics in the treatment of COVID-19, as well as other viral infections (e.g. influenza), is required to prevent antibiotic resistance in accordance with the principles of antimicrobial stewardship.
